# Screening the key genes of hair follicle growth cycle in Inner Mongolian Cashmere goat based on RNA sequencing

**DOI:** 10.5194/aab-63-155-2020

**Published:** 2020-05-26

**Authors:** Rui Su, Gao Gong, Lingtian Zhang, Xiaochun Yan, Fenghong Wang, Lei Zhang, Xian Qiao, Xiaokai Li, Jinquan Li

**Affiliations:** 1 College of Animal Science, Inner Mongolia Agricultural University, Hohhot, Inner Mongolia Autonomous Region, 010018, China; 2 Key Laboratory of Animal Genetics, Breeding and Reproduction, Hohhot, Inner Mongolia Autonomous Region, 010018, China; 3 Key Laboratory of Mutton Sheep Genetics and Breeding, Ministry of Agriculture and Rural Affairs, Hohhot, 010018, China; 4 Engineering Research Center for Goat Genetics and Breeding, Hohhot, Inner Mongolia Autonomous Region, 010018, China

## Abstract

Inner Mongolian Cashmere goat is an excellent local breed selected for the dual-purpose of cashmere and meat. There are three lines of Inner Mongolian Cashmere goat: Erlangshan, Alashan and Aerbasi. Cashmere is a kind of precious textile raw material with a high price. Cashmere is derived from secondary hair follicle (SHF), while hair is derived from primary hair follicle (PHF). The growth cycle of SHF of cashmere goat is 1 year, and it can be divided into three different stages: anagen, catagen and telogen. In this study, we tried to find some important influence factors of SHF growth cycle in skin tissue from Inner Mongolian Cashmere goats by RNA sequencing (RNA-Seq). Three female Aerbasi Inner Mongolian Cashmere goats (2 years old) were used as experimental samples in this study. Skin samples were collected in September (anagen), December (catagen) and March (telogen) at dorsal side from cashmere goats. Results showed that over 511 396 044 raw reads and 487 729 890 clean reads were obtained from sequence data. In total, 51 different expression genes (DEGs) including 29 downregulated genes and 22 upregulated genes were enriched in anagen–catagen comparing group. The 443 DEGs contained 117 downregulated genes and 326 upregulated genes that were enriched in catagen–telogen comparing group. In telogen–anagen comparing group, 779 DEGs were enriched including 582 downregulated genes and 197 upregulated genes. The result of gene ontology (GO) annotation showed that DEGs are in different growth cycle periods, and enriched GO items are mostly related to the transformation of cell and protein. The Kyoto Encyclopedia of Genes and Genomes (KEGG) enrichment result indicated that metabolic process has a great impact on SHF growth cycle. Based on the results of a comprehensive analysis of differentially expressed genes, GO enrichment and KEGG enrichment, we found that *FGF5*, *FGFR1* and *RRAS* had an effect on the hair follicle growth cycle. The results of this study may provide a theoretical basis for further research on the growth and development of SHF in Inner Mongolian Cashmere goats.

## 1 Introduction

China is the largest cashmere producer in the world, with the output of cashmere accounting for about 50 % of the world's total production. In addition, the production of cashmere in Inner Mongolia accounts for about 40 % of the total output of the whole country. Owing to the excellent quality, cashmere from Inner Mongolian Cashmere goats is very expensive and competitive in the textile industry. Inner Mongolian Cashmere goat is a local breed that provides both cashmere and meat, which can be divided into three lines: Erlangshan, Alashan and Aerbasi. These goats live tenaciously in semi-arid steppe of the Inner Mongolia Autonomous Region. Because of the harsh living environment, the fiber diameter of cashmere is usually less than 16 µm, which can keep a body warm in cold winter.

Cashmere is derived from secondary hair follicle (SHF) of cashmere goat, while hair is derived from primary hair follicle (PHF). Hair follicle is a special tissue in the skin which has its own growth cycle and affects the growth of cashmere. A growth cycle mainly consists of three distinct stages: hair follicles begin to develop from growth stage (anagen), stop growing during regression stage (catagen), and then atrophy at rest stage (telogen); finally, hair follicles re-enter a new cycle of growth (Botchkarev et al., 2003 ; Chen et al., 2016; Miao et al., 2016). The growth cycle of hair follicles is influenced by heredity, environment, climate, nutrition and so on. Generally, SHF growth cycle of Inner Mongolian Cashmere goats is 1 year. Li et al. (2008) observed skin tissue sections of Inner Mongolian Cashmere goats for 1 year and concluded that SHF anagen is from April to November, catagen is from December to January, and telogen is from February to March. However, the growth cycle of PHF in cashmere goats is different from that of the SHF. In order to meet the needs of the market, scientific methods were used to select less fineness and higher yield cashmere goats for breeding purposes in order to obtain more high-quality cashmere. With the continuous development of science and technology, molecular breeding will help scientists to speed up the breeding process of Inner Mongolian Cashmere goat.

RNA-Seq is a novel high-throughput sequencing-based approach for global transcriptome mapping, which was first proposed and applied in yeast in 2008 (Nagalakshmi et al., 2008). Transcriptome has temporal and spatial specificity, which means gene expression varies in different tissues or different periods. In the past decade, RNA-Seq technology has been applied in many species, and scientists have developed several methods to analyze these differences that explain some biological phenomena. Researchers have found many important factors affecting the growth cycle of cashmere goats, such as the MAPK signaling pathway, Wnt signal transduction pathway, fibroblast growth factor (FGF) family, bone morphogenetic protein (BMP) family, Notch signal transduction pathway and so on Geng et al. (2013), Wang et al. (2016), and Jin et al. (2016).

In this study, Inner Mongolian Cashmere goat skin samples were sequenced, and the influencing factors and their interactions were explored. By comparing different expression genes (DEGs) among anagen, catagen and telogen, we tried to reveal a fresh viewpoint of growth cycle of hair follicle of cashmere goats. Functional annotation analysis was used to locate influence factors. The real-time quantitative polymerase chain reaction (qRT-PCR) was used to verify the DEGs, and a network diagram of the interaction of various factors was constructed.

## 2 Material and methods

### 2.1 Ethics statement

In this study, skin samples were collected in accordance with the International Guiding Principles for Biomedical Research Involving Animals and were approved by the Animal Ethics Committee of the Inner Mongolia Academy of Agriculture and Animal Husbandry Sciences that is responsible for animal care and use in the Inner Mongolia Autonomous Region of China. In our study, no specific permissions were required for these activities, and the animals did not involve endangered or protected species.

### 2.2 Skin sample preparation for RNA-seq and qRT-PCR validation

Three female Aerbasi Inner Mongolian Cashmere goats at 2 years old from a stud farm (Inner Mongolia Jin Lai Livestock Technology Company, Hohhot, Inner Mongolia) were used in this study. All cashmere goats were raised by feeding practices according to the cashmere goat standard. Skin samples were collected in September (anagen), December (catagen) and March (telogen) of SHF from cashmere goats, the sampling site was the upper one-third of the left scapula along the mid-dorsal and mid-abdominal line. For each goat, we used procaine for local anesthesia to reduce animal pain. After hair shearing and alcohol disinfection, approximately 1 cm${}^{2}$ of skin tissue was grasped with sterile forceps and quickly cut near the tip using sterile scalpel blades. Each clipping was obtained immediately adjacent to the location of the previous shearing. Yunnan Baiyao powder (Yunnan Baiyao Group Co., Ltd., China) was applied immediately to stop the bleeding. Then the samples were quickly put into the liquid nitrogen and finally stored at -80 ${}^{\circ }$C until RNA extraction.

### 2.3 RNA extraction, quantification and qualification

Total RNA was extracted by TRIzol (Invitrogen) under the protocol. In addition, RNA degradation and contamination were monitored on 1 % agarose gels. The purity was checked using the NanoPhotometer^®^ spectrophotometer (IMPLEN, CA, USA). RNA concentration was measured using Qubit^®^ RNA Assay Kit in a Qubit^®^ 2.0 flurometer (Life Technologies, CA, USA). RNA integrity was assessed using the RNA Nano 6000 Assay Kit of the Bioanalyzer 2100 system (Agilent Technologies, CA, USA).

### 2.4 Library preparation for transcriptome sequencing

A total amount of 3 µg of RNA per sample was used as input material for the RNA sample preparations. Sequencing libraries were generated using NEBNext^®^ Ultra™ RNA Library Prep Kit for Illumina^®^ (NEB, USA) following manufacturer's recommendations and index codes were added to attribute sequences to each sample. Briefly, mRNA was purified from total RNA using poly-T oligo-attached magnetic beads. Fragmentation was carried out using divalent cations under elevated temperature in NEBNext First Strand Synthesis Reaction Buffer (5X). First strand complementary DNA (cDNA) was synthesized using random hexamer primer and M-MuLV Reverse Transcriptase (RNase H-). Second strand cDNA synthesis was subsequently performed using DNA Polymerase I and RNase H. Remaining overhangs were converted into blunt ends via exonuclease or polymerase activities. After adenylation of $3^{\prime }$ ends of DNA fragments, NEBNext Adaptor with hairpin loop structure was ligated to prepare for hybridization. In order to select cDNA fragments of preferentially 250∼300 bp in length, the library fragments were purified with AMPure XP system (Beckman Coulter, Beverly, USA). Then 3 µL USER Enzyme (NEB, USA) was used with size-selected, adaptor-ligated cDNA at 37 ${}^{\circ }$C for 15 min followed by 5 min at 95 ${}^{\circ }$C before PCR. Then PCR was performed with Phusion high-fidelity DNA polymerase, Universal PCR primers and Index (X) primer. At last, PCR products were purified (AMPure XP systemc) and library quality was assessed on the Agilent Bioanalyzer 2100 system.

### 2.5 Clustering and sequencing 

The clustering of the index-coded samples was performed on a cBot cluster generation system using TruSeq PE Cluster Kit v3-cBot-HS (Illumina) according to the manufacturer's instructions. After cluster generation, the library preparations were sequenced on an Illumina Hiseq platform and 125 bp or 150 bp paired-end reads were generated.

### 2.6 Data analysis and quality control 

Raw data (raw reads) of fastq format were firstly processed through in-house perl scripts. In this step, clean data (clean reads) were obtained by removing reads containing adapter, reads containing ploy-N and low-quality reads from raw data. At the same time, Q20, Q30 and GC (Q20 and Q30 are Phred scores, which represent sequencing quality, and GC represents the percentage of bases G and C in the sequencing) contents of the clean data were calculated. All the downstream analyses were based on the clean data with high quality.

### 2.7 Reads mapping to the reference genome

Reference genome and gene model annotation files were downloaded from the genome website directly. Index of the reference genome was built using Bowtie v2.2.3 (Langmead et al., 2012) and paired-end clean reads were aligned to the reference genome using TopHat v2.0.12 (Kim et al., 2013). We selected TopHat as the mapping tool because TopHat can generate a database of splice junctions based on the gene model annotation file and thus a better mapping result than other non-splice mapping tools.

### 2.8 Quantification of gene expression level

HTSeq v0.6.1 was used to count the read numbers mapped to each gene. And then the FPKM (Fragments Per Kilobase of transcript per Million mapped reads) of each gene was calculated based on the length of the gene and read count mapped to this gene. FPKM, expected number of Fragments Per Kilobase of transcript sequence per Million base pairs sequenced, considers the effect of sequencing depth and gene length for the read count at the same time, and it is currently the most commonly used method for estimating gene expression levels (Trapnell et al., 2010).

### 2.9 Differential expression analysis

Differential expression analysis of three conditions or groups (three biological replicates per condition) was performed using the DESeq R package (1.18.0) (Wang et al., 2010). DESeq provides statistical routines for determining differential expression in digital gene expression data using a model based on the negative binomial distribution. The resulting P values were adjusted using the approach by Benjamini and Hochberg for controlling the false discovery rate. Genes with an adjusted P value < 0.05 found by DESeq were assigned as differentially expressed.

### 2.10 GO and KEGG enrichment analysis of differentially expressed genes

GO enrichment analysis of differentially expressed genes was implemented by the GOseq R package, in which gene length bias was corrected. GO terms with corrected P value (q value) less than 0.05 were considered significantly enriched by differential expressed genes. KEGG is a database resource for understanding high-level functions and utilities of the biological system, such as the cell, the organism and the ecosystem, from molecular-level information, especially large-scale molecular datasets generated by genome sequencing and other high-throughput experimental technologies (http://www.genome.jp/kegg/, last access: 29 November 2019). We used KOBAS software to test the statistical enrichment of differential expression genes in KEGG pathways.

### 2.11 Validate RNA-seq data and gene expression level

We used qRT-PCR to validate the RNA-seq data and gene expression level in this study. After we extracted total RNA (TRIzol, Invitrogen) from experimental goat skin samples in three periods, we synthesized cDNA (Prime Script™ RT reagent kit with gDNA Eraser Perfect Real Time, TaKaRa) from mRNA. Then, the primers we used were designed and synthesized by Sangon Biotech, depending on the mRNA sequences published on the NCBI database. SYBR Green (TaKaRa) was used in qRT-PCR. β-*actin* acted as internal reference and the DEG expression level was calculated by $2^{-\Delta \Delta \mathrm{ct}}$ (where ct is The threshold cycle) (Livak et al., 2001). Reaction system and condition of the qRT-PCR reaction was based on the protocol. The annealing temperature (TM) was based on the primer design. Results were analyzed by SAS 9.2.

**Figure 1:**
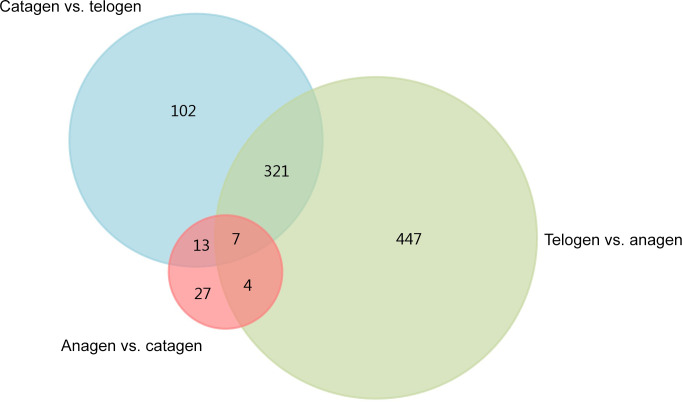
Venn diagram of the construction of DEGs.

**Figure 2:**
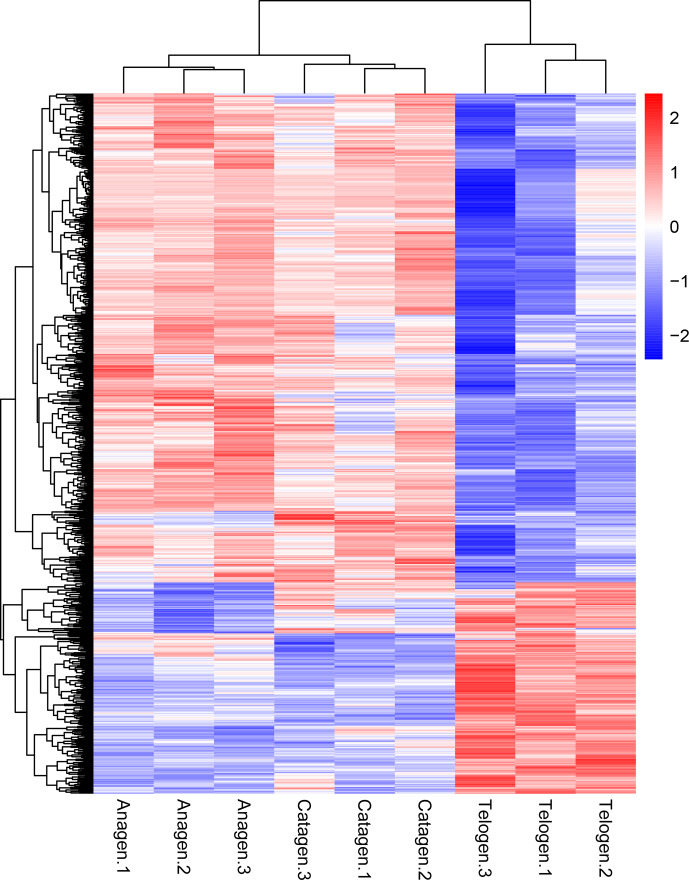
Cluster analysis of all DEGs from the nine sequenced samples. The color scale bar, ranging from blue to red, represents the low-to-high expression, respectively.

## 3 Results

After extracting total RNA from three female Inner Mongolian Cashmere goats in the key stages of anagen, catagen and telogen, we totally constructed nine transcriptome libraries of cashmere goat skin samples and sequenced the RNA. Over 511 396 044 raw reads and 487 729 890 clean reads were obtained in sequence data. Quality control result showed Q20 of each sample was more than 93 % and Q30 more than 85 %, GC content was between 54.77 % and 57.75 % (Table 1). Numbers 1–3 in the sample names represent the 1–3 cashmere goats. Quality control results indicated that the sequencing results were reliable and could be used for subsequent data analysis.

**Table 1:** RNA-Seq quality control result.

Sample name	Raw reads	Clean reads	Clean bases	Error rate (%)	Q20 (%)	Q30 (%)	GC content (%)
anagen-1	51 375 840	48 023 214	7.2G	0.02	95.01	88.6	56.81
anagen-2	54 554 812	51 531 834	7.73G	0.03	93.46	85.53	55.32
anagen-3	58 000 334	56 226 018	8.43G	0.02	95.44	89.34	55.7
catagen-1	58 030 128	55 511 738	8.33G	0.02	95.13	88.74	57.82
catagen-2	59 861 704	57 808 356	8.67G	0.02	95.1	88.59	57.75
catagen-3	69 452 844	66 231 392	9.93G	0.02	95.28	89.2	54.77
telogen-1	43 980 100	41 422 662	6.21G	0.03	93.53	85.64	55.3
telogen-2	42 971 788	41 294 796	6.19G	0.02	95.04	88.54	56.16
telogen-3	58 019 266	55 196 256	8.28G	0.02	95.3	89.11	56.39

After we mapped the clean reads to goat reference genome (*Capra hircus* ARS1), shown in Table 2, we compared each growth cycle stage. There were three comparing groups in our research: (1) anagen to catagen, (2) catagen to telogen and (3) telogen to anagen. By limiting the q value to < 0.05, we found, in total, 51 DEGs including 29 downregulated genes and 22 upregulated genes in the first group. In the second group, there were 443 DEGs in total, containing 117 downregulated genes and 326 upregulated genes. In the third group, there were 779 DEGs including 582 downregulated genes and 197 upregulated genes. In the second group, most DEGs were upregulated, while in the third group downregulated genes play a greater part. A Venn diagram of the construction of DEGs is shown in Fig. 1; it can be seen that the number of DEGs in anagen and telogen is the most, and the number of DEGs in anagen and catagen is the least. These analysis data show that when the hair follicle is in telogen, the gene expression changes greatly compared with other periods. To analyze the expression patterns of genes showing conserved expression between anagen, catagen and telogen, we performed hierarchical clustering to group the genes according to similarities in their patterns of gene expression (Fig. 2). Through hierarchical clustering, it can be found that anagen and catagen are clustered together, and the gene expression patterns are similar, while the gene expression patterns of telogen are quite different from those of anagen and catagen.

**Table 2:** RNA-Seq mapping result.

Sample name	anagen-1	anagen-2	anagen-3	catagen-1	catagen-2	catagen-3	telogen-1	telogen-2	telogen-3
Total reads	48 023 214	51 531 834	56 226 018	55 511 738	57 808 356	66 231 392	41 422 662	41 294 796	55 196 256
Total mapped	42 896 370 (89.32 %)	45 416 889 (88.13 %)	50 625 023 (90.04 %)	49 484 915 (89.14 %)	51 760 940 (89.54 %)	58 988 206 (89.06 %)	36 481 485 (88.07 %)	37 061 986 (89.75 %)	48 588 674 (88.03 %)
Multiple mapped	1 469 258 (3.06 %)	1 794 744 (3.48 %)	1 766 143 (3.14 %)	1 694 442 (3.05 %)	1 858 878 (3.22 %)	1 971 520 (2.98 %)	1 108 880 (2.68 %)	1 119 638 (2.71 %)	1 532 100 (2.78 %)
Uniquely mapped	41 427 112 (86.26 %)	43 622 145 (84.65 %)	48 858 880 (86.9 %)	47 790 473 (86.09 %)	49 902 062 (86.32 %)	57 016 686 (86.09 %)	35 372 605 (85.39 %)	35 942 348 (87.04 %)	47 056 574 (85.25 %)
Reads map to “+”	20 664 114 (43.03 %)	21 751 032 (42.21 %)	24 380 196 (43.36 %)	23 863 999 (42.99 %)	24 909 939 (43.09 %)	28 478 781 (43 %)	17 648 586 (42.61 %)	17 956 239 (43.48 %)	23 511 002 (42.6 %)
Reads map to “-”	20 762 998 (43.24 %)	21 871 113 (42.44 %)	24 478 684 (43.54 %)	23 926 474 (43.1 %)	24 992 123 (43.23 %)	28 537 905 (43.09 %)	17 724 019 (42.79 %)	17 986 109 (43.56 %)	23 545 572 (42.66 %)
Non-splice reads	25 215 457 (52.51 %)	27 150 222 (52.69 %)	31 834 685 (56.62 %)	29 077 447 (52.38 %)	30 290 201 (52.4 %)	36 291 421 (54.79 %)	21 375 623 (51.6 %)	22 539 791 (54.58 %)	31 783 754 (57.58 %)
Splice reads	16 211 655 (33.76 %)	16 471 923 (31.96 %)	17 024 195 (30.28 %)	18 713 026 (33.71 %)	19 611 861 (33.93 %)	20 725 265 (31.29 %)	13 996 982 (33.79 %)	13 402 557 (32.46 %)	15 272 820 (27.67 %)

**Figure 3:**
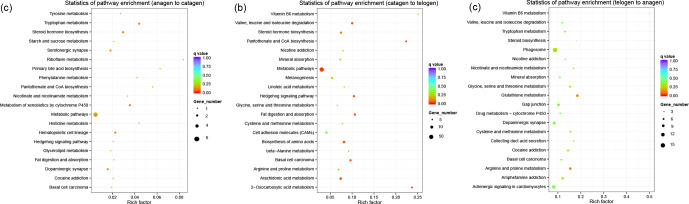
KEGG pathway analysis of DEGs. **(a)** DEGs between anagen and catagen. **(b)** DEGs between catagen and telogen. **(c)** DEGs between telogen and anagen.

After comparing the DEGs between each group, GO annotations were analyzed (see the Supplement). In the first group, we found, for biological process, that upregulated DEGs were mostly enriched in fatty acid beta-oxidation using acyl-CoA dehydrogenase (GO:0033539), acute inflammatory response (GO:0002526) and acute-phase response (GO:0006953); downregulated DEGs were mostly enriched in regulation of cell shape (GO:0008360), injection of substances into other organism (GO:0035737) and envenomation, resulting in modification of morphology or physiology of other organisms (GO:0035738). Interestingly, both up- and downregulated DEGs were enriched in protein complex assembly (GO:0006461) and protein complex biogenesis (GO:0070271). For the cellular component, both up- and downregulated DEGs were mainly enriched in cytoskeletal part (GO:0044430), cytoskeleton (GO:0005856), intermediate filament (GO:0005882) and intermediate filament cytoskeleton (GO:0045111). Then, for molecular function, most DEGs were enriched in metal ion binding (GO:0046872) and cation binding (GO:0043169). RNA-directed RNA polymerase activity (GO:0003968), RNA polymerase activity (GO:0034062) and serine-type endopeptidase activity (GO:0004252) only have upregulated DEGs. In the second group that has DEGs between catagen and telogen, upregulated DEGs enriched in protein import into nucleus (GO:0006606) in biological process, intermediate filament (GO:0005882) and dynein binding (GO:0045502). A few downregulated DEGs were enriched in cytoplasmic transport (GO:0016482) in biological process, intracellular non-membrane-bounded organelle (GO:0043232) in cellular component and dynein binding (GO:0045502) in molecular function. Finally, in the third group DEGs were mainly enriched in protein import into nucleus (GO:0006606) in biological process, intermediate filament (GO:0005882) in cellular component and dynein binding (GO:0045502) in molecular function. These results showed that DEGs are in different growth cycle periods and GO items mostly related to the transformation of cell and protein.

**Figure 4:**
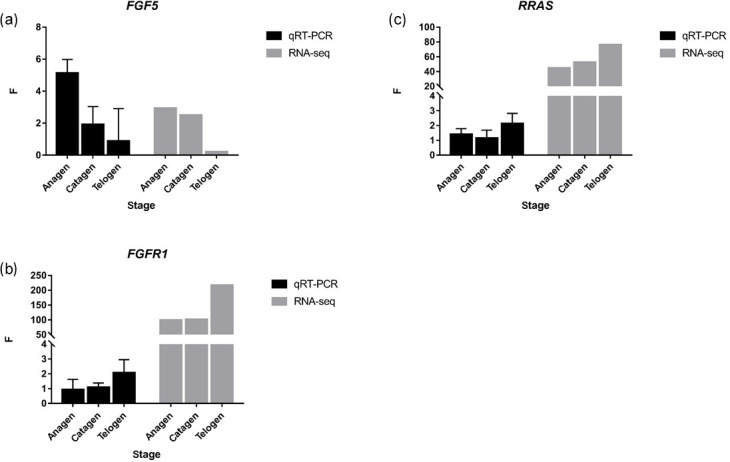
*FGF5*, *FGFR1* and *RRAS* were detected by qRT-PCR, and the trend was similar to RNA-seq. ${}^{**}$ p value < 0.01, ${}^{*}$ p value < 0.05.

In order to collect the molecular interaction, reaction and relation of the DEGs of the three groups, the KEGG pathway was also analyzed. DEGs in these three groups were mainly enriched in the pathways related to metabolism (Fig. 3), while most DEGs in the first and second groups were enriched in the metabolic pathway (chx01100). However, most DEGs in third group were enriched in phagosome (chx04145). The results of KEGG enrichment indicated that the metabolic pathway had a great impact on SHF growth cycle.

**Figure 5:**
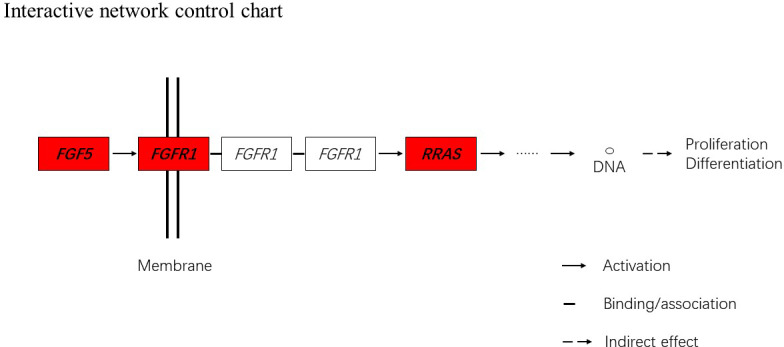
Interactive network control chart. We find that *FGF5* may influence *FGFRL1* and can directly regulate and then indirectly affect *RRAS*, ultimately affecting proliferation and differentiation.

Together with DEGs, GO and KEGG analysis data, we identified three genes including fibroblast growth factor 5(*FGF5*), fibroblast growth factor receptor 1(*FGFR1*) and RAS related (*RRAS*), which may be related to hair follicle growth and development in MAPK signaling pathway and verified their expression level by qRT-PCR. The primer sequences information is shown in Table 3. Results showed the expression trend in qRT-PCR of these three genes was basically the same with RNA-Seq (Fig. 4). It can be seen that the expression trend of *FGF5* is opposite to that of *FGFR1* and *RRAS*. It is possible that there is a negative regulatory relationship between *FGF5* and *FGFR1*, while a positive regulatory relationship between *FGFR1* and *RRAS*. *FGF5* was highly expressed in anagen and expressed lower in telogen. *FGFR1* was different from *FGF5*, while telogen expressed higher and anagen expressed lower. Depending on the expression of genes and MAPK pathway, we can draw an interactive network control chart (Fig. 5). The interactive network control chart showed that *FGF5*, *FGFR1* and *RRAS* were located at essential places in the MAPK signal pathway. Firstly, together with other factors, *FGF5* directly activated *FGFR1* which was a receptor on membrane. *FGFR1* may combine with *GRB2* and *SOS*, then activate *RRAS*. Finally, after a series of activation and phosphorylation interactions, cell proliferation and differentiation were affected and then regulated the periodic growth of hair follicles.

**Table 3:** Primer sequence in qRT-PCR.

Gene name		Sequence	Product length (bp)	TM (${}^{\circ }$C)
β-*actin*	F:	GGCAGGTCATCACCATCGG	158	60
	R:	CGTGTTGGCGTAGAGGTCTTT		
*FGF5*	F:	CAGAGTGGGCATCGGTTTC	163	58
	R:	TATTCCTACAATCCCCTGAGACA		
*FGFR1*	F:	TGACCTCGCCGCTGTACCTG	113	64
	R:	GCTCTTCTTCGTGCCGCTCTTC		
*RRAS*	F:	GCTGACCATCCAGTTCATCCAGTC	81	63
	R:	GCGCAGATCTTCGTGTAGGAGTC		

## 4 Discussion

Since the 1960s, researchers tried to explore the periodic changes in SHF in cashmere goats, from phenotype to the molecular mechanism. The expression gene at RNA level varies in time and tissue, so transcriptome sequencing is a direct way to explore gene expression changes. Therefore, we studied the growth cycle of SHF in cashmere goats from the perspective of transcriptome. In a recent study, we chose hair follicle, pulled out from the dorsal side, as experimental samples to analyze the SHF and PHF in catagen and telogen growth cycles of cashmere goats, while the effect of other factors were excluded. We identified a set of differentially expressed known and novel genes in hair follicles, such as STC2, ROR2 and VEGFA, which may be related to hair cycle growth and other physiological functions (Su et al., 2018). However, in a recent study, researchers found that stem cells in hair follicle were regulated by the intra-follicle adjacent micro-environmental niche, while this niche was also modulated dynamically by extra-follicular macro-environmental signals (Chen et al., 2016). Therefore, we used skin in anagen, catagen and telogen as sample to sequence the transcriptome in this study.

Geng et al. (2013) studied the hair follicle development and cycling of Shaanbei white cashmere goat. It was found that a large number of DEGs were mainly related to the cellular process, cell and cell part, binding, biological regulation and metabolic process among the different stages of hair follicle development. In addition, Wnt, Shh, TGF-β and Notch signal pathways may be involved in the development of hair follicles (Geng et al., 2013). In this study, there were 51 DEGs between anagen and catagen, 443 DEGs between catagen and telogen, and 779 DEGs between telogen and anagen. The most DEGs were gained between telogen and anagen, while fewest DEGs were between anagen and catagen. This may mean that, from growth stage to resting phase of hair follicle, changes were gradually taking place inside the skin. In addition, in hair follicle going from resting phase into a new round of cycle growth phase, the internal molecular microstate of the skin has undergone a great change. We also found that upregulated genes between catagen and telogen played a great count, while downregulated genes accounted for the majority between telogen and anagen. This may indicate that the expression level of most genes in skin was consistent with the hair follicle activity.

The mechanism of SHF growth of cashmere goats is still in the exploratory stage. Hair follicle growth and development is an extremely complicated process, and it is influenced by many internal and external factors. It has been reported that hormone level, light duration and nutrition, as well as some other important factors, had an important effect on the growth and development of hair follicles in cashmere goats. Melatonin is one of the most important hormones that affects the growth and development of SHF in cashmere goats. It could promote the initiation and maturation of secondary follicles and increased their population, while the beneficial effect of melatonin on secondary follicle population remained throughout the cashmere goat's whole life (Yang et al., 2019). Recently, the mechanism of melatonin effect on SHF of cashmere goats was reported. Included in the enhancement of activities of antioxidant enzymes are, for example, superoxide dismutase and glutathione peroxidase (GSH- Px), elevated total antioxidant capacity, upregulated anti-apoptotic Bcl-2 expression, and downregulated expression of the pro-apoptotic proteins, Bax and caspase-3 (Yang et al., 2019). Daily light exposure also played a large role in SHF growth. Exposure to a short photoperiod extended the anagen phase of the cashmere goat hair follicle to increase cashmere production. Assessments of tissue sections indicated that the short photoperiod significantly induced cashmere growth (Liu et al., 2016). When reducing the daily light exposure for cashmere goats to 7 h, the SHF activity and the melatonin concentration in July and the cashmere fiber length and fiber weight in October were significantly increased compared to natural daily photoperiod (Zhang et al., 2019). From a nutritional aspect, researchers found that there was a tendency or a significant interaction effect of Cu and Mo on cashmere growth (P=0.076) or diameter (P<0.05), which might be accomplished by changing the number of secondary follicle and active secondary follicle, as well as secondary-to-primary follicle ratio (Zhang et al., 2011). Researchers also found that dietary supplement of Essential Oils-Cobalt (EOC) significantly promoted cashmere goat hair fiber quality (P<0.05) (Lei et al., 2018). Because the growth and development of SHF of cashmere goats are a very complex process, many scientists are still exploring the important factors it affects and its growth and development mechanism.

In this study, the expression of some genes from the MAPK signal pathway was different in different periods. Therefore, it can be predicted that these DEGs would have an impact on cell proliferation and differentiation through the MAPK signal pathway, and then it affected the growth and development of hair follicles. Previous studies found the MAPK signal pathway was an important pathway for the growth and development of hair follicle in mammal. Akilli Öztürk et al. demonstrated an essential role of Gab1 upstream of MAPK in the regulation of the hair cycle and the self-renewal of hair follicle stem cells in mouse (Akilli Öztürk et al., 2015). Liu et al. found inhibition of MAPK-ERK-Mfn2 axis abrogated the protective effects of *Sirt1* on hair follicle stem cell survival, migration and proliferation (Liu et al., 2018). Zhang et al. analyzed the transcriptome of cashmere goat and milk goat and discovered that the MAPK signal pathway was involved in hair follicle cycling in both cashmere and milk goat (Zhang et al., 2020). Jin et al. suggested that *LAMTOR3* influences the character of cashmere fiber, and it might regulate the development of hair follicle and cashmere growth by inducing the MAPK signaling pathway (Jin et al., 2019). Platelet-rich plasma (PRP) was an innovative treatment of androgenic alopecia in the early stages of development; PRP might promote proliferation of dermal papilla cells by activated MAPK and Akt signal pathways (Xiao et al., 2019). Recently, researchers found that the MAPK signal pathway not only affected hair follicle growth and development but also affected poultry feather growth and development. Fang et al. revealed that the altered genes or targets of altered miRNAs were involved in multiple biological processes and pathways, including the MAPK signal pathway that related to feather growth and development (Fang et al., 2018). This research all demonstrated MAPK signal pathway's importance in hair follicle growth and development. Our study indicated that *FGF5*, *FGFRL1* and *RRAS* influenced the growth and development of hair follicle in Inner Mongolian Cashmere goat through MAPK signal pathway. This will provide a theoretical basis for further research on the growth and development of SHF in Inner Mongolian Cashmere goats.

## 5 Conclusions

In this study, we tried to find some important influencing factors of SHF growth cycle in skin tissue from Inner Mongolian Cashmere goats by RNA-Seq. As results, over 511 396 044 raw reads and 487 729 890 clean reads were obtained from sequence data. In total, 51 DEGs including 29 downregulated genes and 22 upregulated genes were enriched between anagen and catagen. After comparing catagen to telogen, we got 443 DEGs in total containing 117 downregulated genes and 326 upregulated genes. In the telogen–anagen comparing group, 779 DEGs including 582 downregulated genes and 197 upregulated genes were found. DEGs were annotated in different growth cycle periods by GO analysis in each comparing group. The GO items were mostly related to the transformation of cell and protein. KEGG enrichment result indicated that the metabolic process had a great impact on SHF growth cycle. Comprehensive analysis results of DEGs, GO enrichment and KEGG enrichment that our study excavated indicated that *FGF5*, *FGFRL1* and *RRAS* influenced the growth and development of hair follicle in Inner Mongolian Cashmere goats through MAPK signal pathway.

## Supplement

10.5194/aab-63-155-2020-supplement The supplement related to this article is available online at: https://doi.org/10.5194/aab-63-155-2020-supplement.

## Data Availability

All data files are available from the SRA database (, last access: 18 May 2020, accession number SUB6509124; Gong, 2020).
