# Supplementary material for: Screening the key genes of hair follicle growth cycle in Inner Mongolian Cashmere goat based on RNA sequencing
Source: Arch Anim Breed. 2020 May 26;63(1):155–64. doi: 10.5194/aab-63-155-2020 (PMC7256851; doi:10.5194/aab-63-155-2020)
Supplement: The supplement related to this article is available online at: https://doi.org/10.5194/aab-63-155-2020-supplement. [file aab-63-155-supplement.zip › aab-63-155-2020-supplement-title-page.pdf]

Supplement of Arch. Anim. Breed., 63, 155–164, 2020
https://doi.org/10.5194/aab-63-155-2020-supplement
© Author(s) 2020. This work is distributed under
the Creative Commons Attribution 4.0 License.

Open Access
Archives Animal Breeding

Supplement of

Screening the key genes of hair follicle growth cycle in Inner Mongolian Cashmere goat based on RNA sequencing

Rui Su et al.

Correspondence to: Jinquan Li (lijinquan_nd@126.com)

- aab-63-155-2020-supplement-title-page.pdf
- supplement files
  - GO anagen to catagen.pdf
  - GO catagen to telogen.pdf
  - GO telogen to anagen.pdf

The copyright of individual parts of the supplement might differ from the CC BY 4.0 License.
