# Supplementary material for: Screening the key genes of hair follicle growth cycle in Inner Mongolian Cashmere goat based on RNA sequencing
Source: Arch Anim Breed. 2020 May 26;63(1):155–64. doi: 10.5194/aab-63-155-2020 (PMC7256851; doi:10.5194/aab-63-155-2020)
Supplement: The supplement related to this article is available online at: https://doi.org/10.5194/aab-63-155-2020-supplement. [file aab-63-155-supplement.zip › supplement files/GO catagen to telogen.pdf]

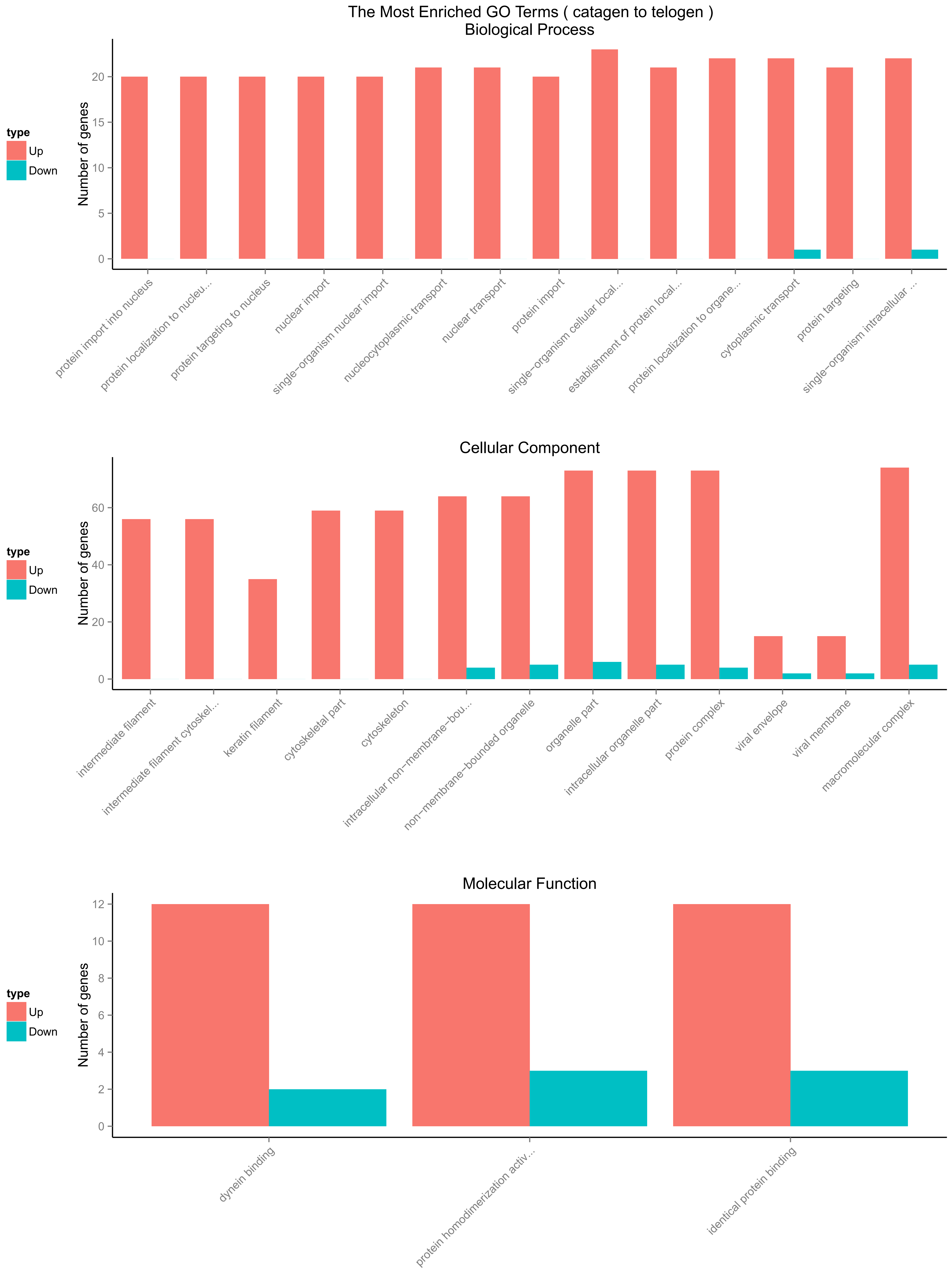

The Most Enriched GO Terms (catagen to telogen)
Biological Process
Number of genes
type
Up
Down
protein import into nucleus
protein localization to nucleu...
protein targeting to nucleus
nuclear import
single−organism nuclear import
nucleocytoplasmic transport
nuclear transport
protein import
single−organism cellular local...
establishment of protein local...
protein localization to organe...
cytoplasmic transport
protein targeting
single−organism intracellular ...
Cellular Component
intermediate filament
intermediate filament cytoskel...
keratin filament
cytoskeletal part
cytoskeleton
intracellular non−membrane−bou...
non−membrane−bounded organelle
organelle part
intracellular organelle part
protein complex
viral envelope
viral membrane
macromolecular complex
Molecular Function
dynein binding
protein homodimerization activ...
identical protein binding
