# Supplementary material for: Screening the key genes of hair follicle growth cycle in Inner Mongolian Cashmere goat based on RNA sequencing
Source: Arch Anim Breed. 2020 May 26;63(1):155–64. doi: 10.5194/aab-63-155-2020 (PMC7256851; doi:10.5194/aab-63-155-2020)
Supplement: The supplement related to this article is available online at: https://doi.org/10.5194/aab-63-155-2020-supplement. [file aab-63-155-supplement.zip › supplement files/GO anagen to catagen.pdf]

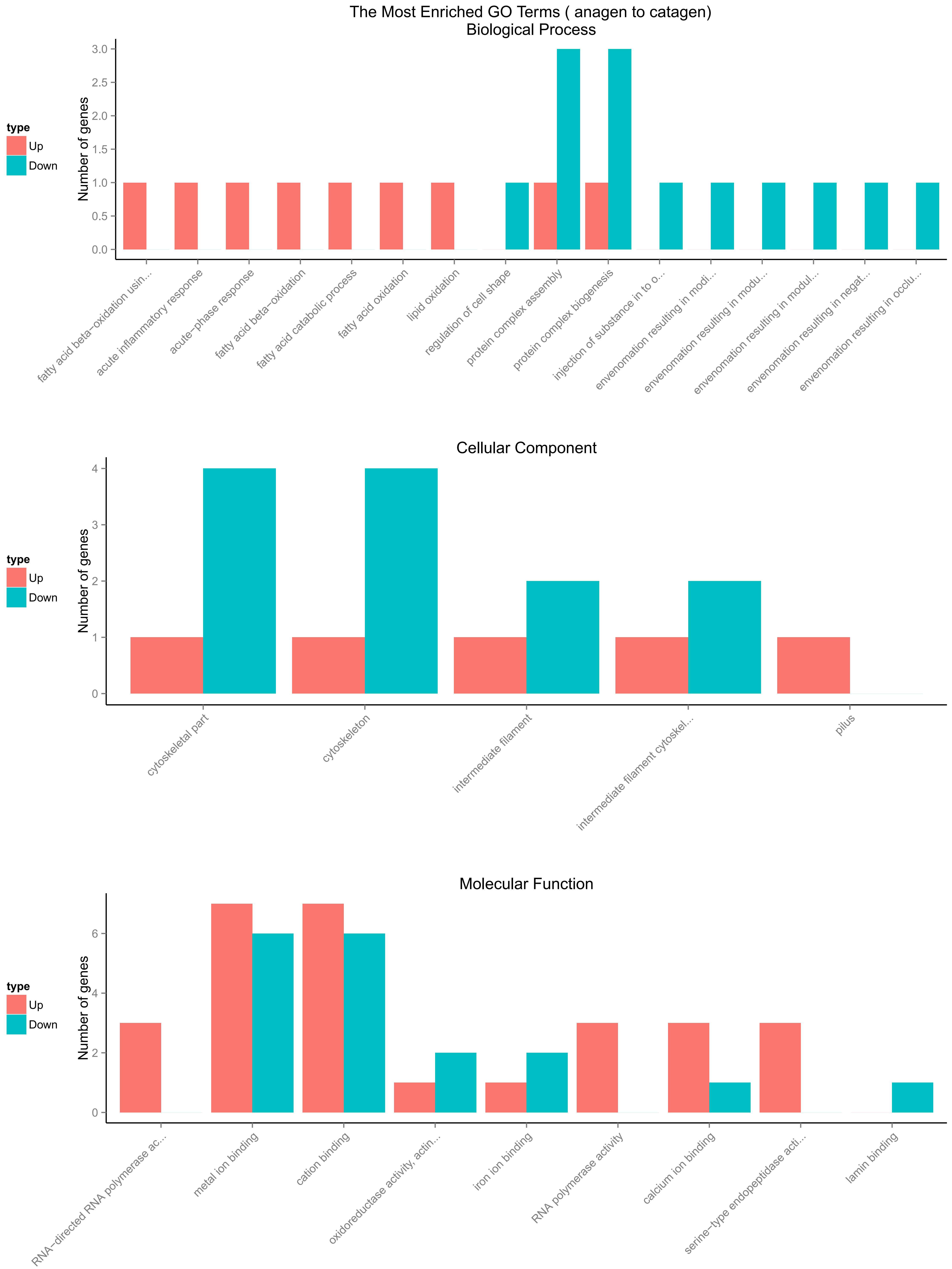

The Most Enriched GO Terms (anagen to catagen)
Biological Process
Number of genes
type
Up
Down
fatty acid beta−oxidation usin...
acute inflammatory response
acute−phase response
fatty acid beta−oxidation
fatty acid catabolic process
fatty acid oxidation
lipid oxidation
regulation of cell shape
protein complex assembly
protein complex biogenesis
injection of substance in to o...
envenomation resulting in modi...
envenomation resulting in modu...
envenomation resulting in modul...
envenomation resulting in negat...
envenomation resulting in occlu...
Cellular Component
cytoskeletal part
cytoskeleton
intermediate filament
intermediate filament cytoskel...
pilus
Molecular Function
RNA−directed RNA polymerase ac...
metal ion binding
cation binding
oxidoreductase activity, actin...
iron ion binding
RNA polymerase activity
calcium ion binding
serine−type endopeptidase acti...
lamin binding
