# Supplementary material for: Screening the key genes of hair follicle growth cycle in Inner Mongolian Cashmere goat based on RNA sequencing
Source: Arch Anim Breed. 2020 May 26;63(1):155–64. doi: 10.5194/aab-63-155-2020 (PMC7256851; doi:10.5194/aab-63-155-2020)
Supplement: The supplement related to this article is available online at: https://doi.org/10.5194/aab-63-155-2020-supplement. [file aab-63-155-supplement.zip › supplement files/GO telogen to anagen.pdf]

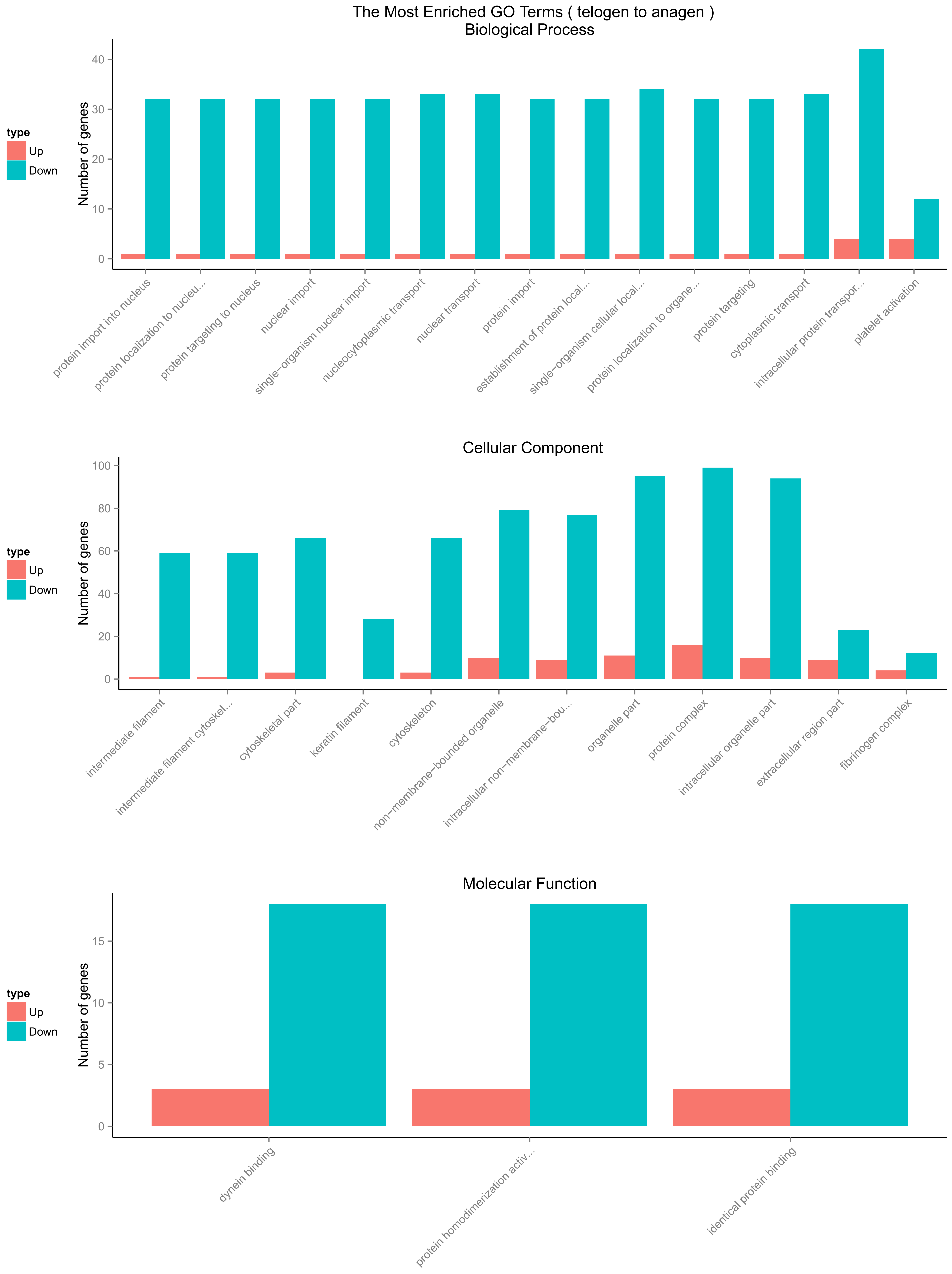
The Most Enriched GO Terms ( telogen to anagen )
Biological Process
Number of genes
type
Up
Down
protein import into nucleus
protein localization to nucleu...
protein targeting to nucleus
nuclear import
single−organism nuclear import
nucleocytoplasmic transport
nuclear transport
protein import
establishment of protein local...
single−organism cellular local...
protein localization to organe...
protein targeting
cytoplasmic transport
intracellular protein transpor...
platelet activation
Cellular Component
intermediate filament
intermediate filament cytoskel...
cytoskeletal part
keratin filament
cytoskeleton
non−membrane−bounded organelle
intracellular non−membrane−bou...
organelle part
protein complex
intracellular organelle part
extracellular region part
fibrinogen complex
Molecular Function
dynein binding
protein homodimerization activ...
identical protein binding
